# Mechanical forces induce an asthma gene signature in healthy airway epithelial cells

**DOI:** 10.1038/s41598-020-57755-8

**Published:** 2020-01-22

**Authors:** Ayşe Kılıç, Asher Ameli, Jin-Ah Park, Alvin T. Kho, Kelan Tantisira, Marc Santolini, Feixiong Cheng, Jennifer A. Mitchel, Maureen McGill, Michael J. O’Sullivan, Margherita De Marzio, Amitabh Sharma, Scott H. Randell, Jeffrey M. Drazen, Jeffrey J. Fredberg, Scott T. Weiss

**Affiliations:** 1 Channing Division of Network Medicine, Department of Medicine, Brigham and Women’s Hospital, Boston, MA USA; 2 Department of Physics, Northeastern University, Boston, MA USA; 3 Program in Molecular Integrative Phyisological Sciences, Department of Environmental Health, Harvard TH Chan School of Public Health, Boston, MA USA; 4 Computational Health Informatics Program, Boston Children’s Hospital, Boston, MA USA; 5 Centre for Research and Interdisciplinarity (CRI), Paris, F-75014 France; 6 Genomic Medicine Institute, Lerner Research Institute, Cleveland Clinic, Cleveland, OH 44195 USA; 7 Department of Molecular Medicine, Cleveland Clinic Lerner College of Medicine, Case Western Reserve University, Cleveland, OH 44195 USA; 8 Case Comprehensive Cancer Center, Case Western Reserve University School of Medicine, Cleveland, Ohio 44106 USA; 9 Marsico Lung Institute/Cystic Fibrosis Center, University of North Carolina, Chapel Hill, NC USA

**Keywords:** Transcriptomics, Systems biology

## Abstract

Bronchospasm compresses the bronchial epithelium, and this compressive stress has been implicated in asthma pathogenesis. However, the molecular mechanisms by which this compressive stress alters pathways relevant to disease are not well understood. Using air-liquid interface cultures of primary human bronchial epithelial cells derived from non-asthmatic donors and asthmatic donors, we applied a compressive stress and then used a network approach to map resulting changes in the molecular interactome. In cells from non-asthmatic donors, compression by itself was sufficient to induce inflammatory, late repair, and fibrotic pathways. Remarkably, this molecular profile of non-asthmatic cells after compression recapitulated the profile of asthmatic cells before compression. Together, these results show that even in the absence of any inflammatory stimulus, mechanical compression alone is sufficient to induce an asthma-like molecular signature.

## Introduction

Bronchial epithelial cells (BECs) form a physical barrier that protects pulmonary airways from inhaled irritants and invading pathogens^1,2^. Moreover, environmental stimuli such as allergens, pollutants and viruses can induce constriction of the airways^3^ and thereby expose the bronchial epithelium to compressive mechanical stress. In BECs, this compressive stress induces structural, biophysical, as well as molecular changes^4,5^, that interact with nearby mesenchyme^6^ to cause epithelial layer unjamming^1^, shedding of soluble factors, production of matrix proteins, and activation matrix modifying enzymes, which then act to coordinate inflammatory and remodeling processes^4,7–10^.

Growing evidence supports the notion that mechanical stress in the airway induces not only developmental, homeostatic and reparative responses in the healthy lung but also pathophysiologic processes in the asthmatic lung^2,11–13^. For example, mechanical stimuli induce early inflammatory and remodeling factors^6,7,14,15^, microRNAs (miRs)^16,17^, and cell proliferation^18^. Using protein-protein interaction networks, here we compare responses to compressive stress in human BECs (HBECs) obtained from non-asthmatic versus asthmatic donors. Our hypothesis was, that using a systems biology approach to examine the effect of mechanical forces acting on structural cells in the airway, will elucidate the complex transcriptional programs that contribute to disease development and pathophysiology.

## Results

### Uncompressed asthmatic HBECs express a distinct gene signature

**Figure 1:**
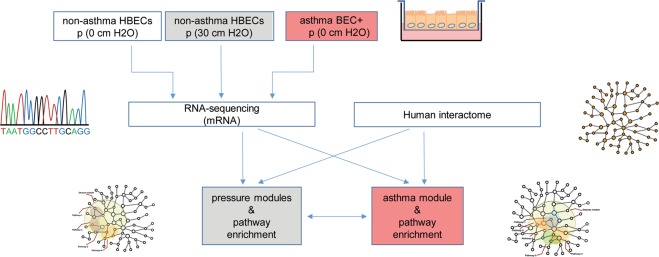
Workflow for the analysis of compression induced pathological signatures in HBECs from non-asthmatic donors. HBECs from non-asthmatic donors were compressed and the mRNA expression was detected by RNA-sequencing. Using the human PPI, the compression modules were defined. Pathway enrichment analysis was performed in both, compressed cells and asthmatic HBECs, both normalized to healthy non-asthmatic HBECs.

There is an ongoing debate whether the development of asthma arises from early disruption of the airway epithelium or imbalance of immune responses^19^. To investigate the former possibility, we used both non-asthmatic and asthmatic HBECs grown in air-liquid interface (ALI) culture in the absence of immune cells or stimuli. We combined RNA-sequencing and the human protein-protein interaction network to identify relevant genes and pathways (Fig. 1).

**Figure 2:**
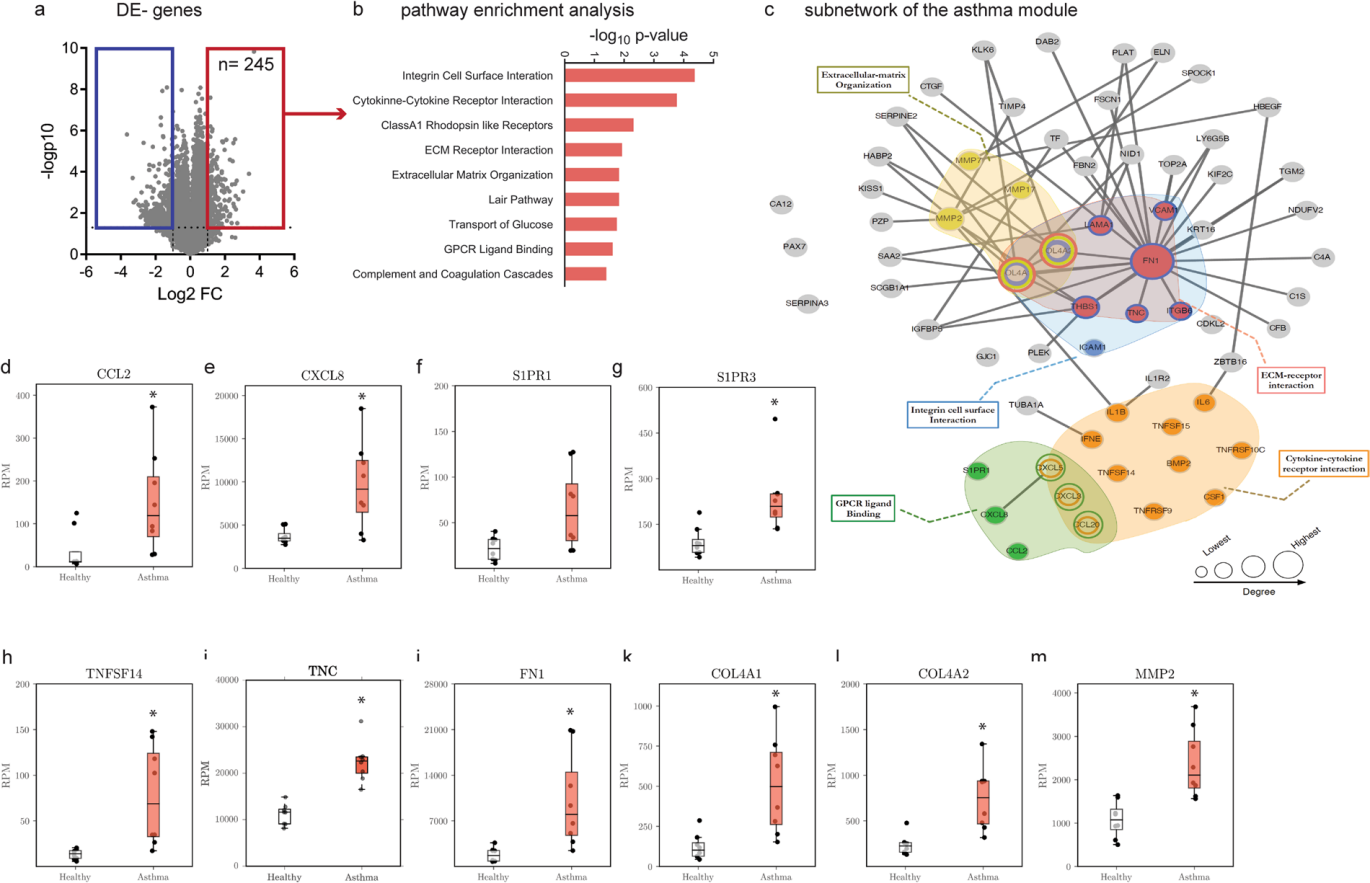
Asthma signature in HBECs. (**a**) Volcano plot representation of gene expression changes between healthy non-asthmatic and asthmatic HBECs. **(b)** Pathway enrichment analysis for genes upregulated in the asthmatic HBECs compared to non-asthmatic HBECs. **(c)** A subnetwork of the asthma disease module is shown. Activated pathways are highlighted in colours. RNA expression of genes promoting inflammation, including the chemokines **(d)** Ccl2, **(e)** Cxcl8, sphingosine-1-phosphate receptors **(f)** S1PR1, **(g)** S1PR3 as well as the secreted protein **(h)** TNFSF14. Asthmatic HBECs express elevated remodeling associated factors, including **(i)** tenascin (TNC), **(j)** fibronectin 1(FN1), **(k)** collagen 4 chain Col4a1 and **(l)** matrix metalloproteinase MMP2. **(m)** Venn diagram summarizes the gene overlap between the asthma module and publicly available epithelial derived datasets deposited under GSE63142 and GSE37693. Values summarize the expression levels for n = 8 independent samples per group. The box and whisker plots represent the minimum, 25th percentile, median, 75th percentiles and the maximum. *p < 0.05, to control was considered significant.

Initial analysis indicated that 774 genes were differentially expressed (DE) between non-asthmatic and asthmatic HBECs, with 254 being upregulated and 520 downregulated (Fig. 2a). A network approach, using gene expression data levels^20^ then identified a neighborhood of DE genes between non-asthmatic and asthmatic HBECs, that formed a significantly connected module in the PPI network and defined a disease signature, which we termed the “asthma module” (see methods section for details). Genes mapping to this asthma module were validated with available literature, ascertaining the significance of the identified gene list. Pathway analysis revealed the increased expression of genes belonging to G-protein coupled receptor (GPCR) ligand binding (p = 2.24E-02)^21–26^, integrin cell surface interaction (p = 4.25E-05), cytokine - cytokine receptor interaction (p = 1.70E-04), extracellular matrix (ECM) receptor interaction (p = 1.18E-02) and ECM organization (p = 1.49E-02) were enriched (Fig. 2b and Table 1). Since a disease module usually comprises several hundred genes, we visualized the upregulated genes and the pathways that they mapped to in a subnetwork entailing inflammatory and remodeling mechanisms (Fig. 2c). The GPCR pathway included the chemokines *CCL2*^21^, *CCL20*^22^, *CXCL3*^23^, *CXCL5*^24^, *CXCL8*^24^ (Interleukin (IL)-8) and sphingosine 1-phosphate receptors *S1PR1* and *S1PR3*^25^ (Fig. 2d–g). The cytokine - cytokine receptor interaction pathway comprised chemokines as well as several cytokines and growth factors, including *IL-6*^26^ and *IL-1β*^27^. Genes coding for matrix proteins as well as matrix modifying enzymes mapped to the pathways associated with matrix interaction and remodeling. The expression of the most central gene fibronectin 1 (*FN1*) and *IL-8* as a peripheral gene were validated by real-time RT-PCR in independent samples (Supplementary Fig. S2). In asthma, these pathways regulate recruitment of inflammatory cells as well as remodeling processes (Fig. 2h–m). To further validate and confirm above listed results, the overlap with previously described and published asthma data sets (see methods section) was assessed. A highly significant overlap was found with ALI-grown healthy epithelial cells exposed to interleukin (IL) -13 (GSE37693; p = 1.61E-36) and freshly isolated epithelial cells from severe asthmatic patients (GSE63142; p = 1.24E-08) (Fig. 2m).

**Table 1:** Active pathways in asthmatic HBECs compared to healthy HBEC at baseline.

	Pathway	adj. p-value^a^ (Bonferroni)	genes in pathway
1	Integrin cell surface interaction	4.25E-05	COL4A2, ICAM1, COL4A1, LAMA1, ITGB6, THBS1, TNC, VCAM1, FN1
2	Cytokine- cytokine- receptor interaction	1.70E-04	CXCL8, IL1B, CCL2, TNFRSF9, CXCL3, CXCL5, IL6, IFNE, TNFSF14, TNFSF15, CSF1, BMP2, TNFRSF10C, CCL20
3	Class A1 Rhodopsin receptor interaction	4.74E-03	CCL2, CXCL3, CXCL8, HTR7, S1PR1, AGT, EDNRA, MTNR1A, GPR68, CXCL5, S1PR3, NPBWR1, CCL20
4	Platelet amyloid precursor protein pathway	6.39E-03	SERPINE1, COL4A2, PLAT, COL4A1
5	ECM receptor interaction	1.18E-02	COL4A2, FN1, COL4A1, LAMA1, ITGB6, THBS1, TNC
6	Extracellular matrix organization	1.49E-02	COL4A2, COL4A1, MMP2, TLL2, MMP7, MMP17, COL22A1
7	local acute inflammatory response pathway	1.49E-02	VCAM1, CXCL8, ICAM1, IL6
8	Transport of glucose and other sugars, bile, salts, acids, metal ion, and amine compounds	1.73E-02	SLC22A3, SLC13A5, SLC39A8, SLC2A3, RHCG, SLC39A2, SLC6A12
9	GPCR ligand binding	2.24E-02	CCL2, CXCL3, CXCL8, HTR7, S1PR1, AGT, ENDRA, FZD7, MTNR1A, GPR68, CXCL5, S1PR3, NPBWR1, CCL20
10	Cartilage oligomeric matrix protein pathway	3.99E-02	C1S, C1R, CFB, C4A

^a^Adjusted p-value: p-value was calculated using the Fisher exact test and adjustment was done using Bonferroni correction.

### Compressive stress induces pronounced molecular changes in bronchial epithelial cells

**Figure 3:**
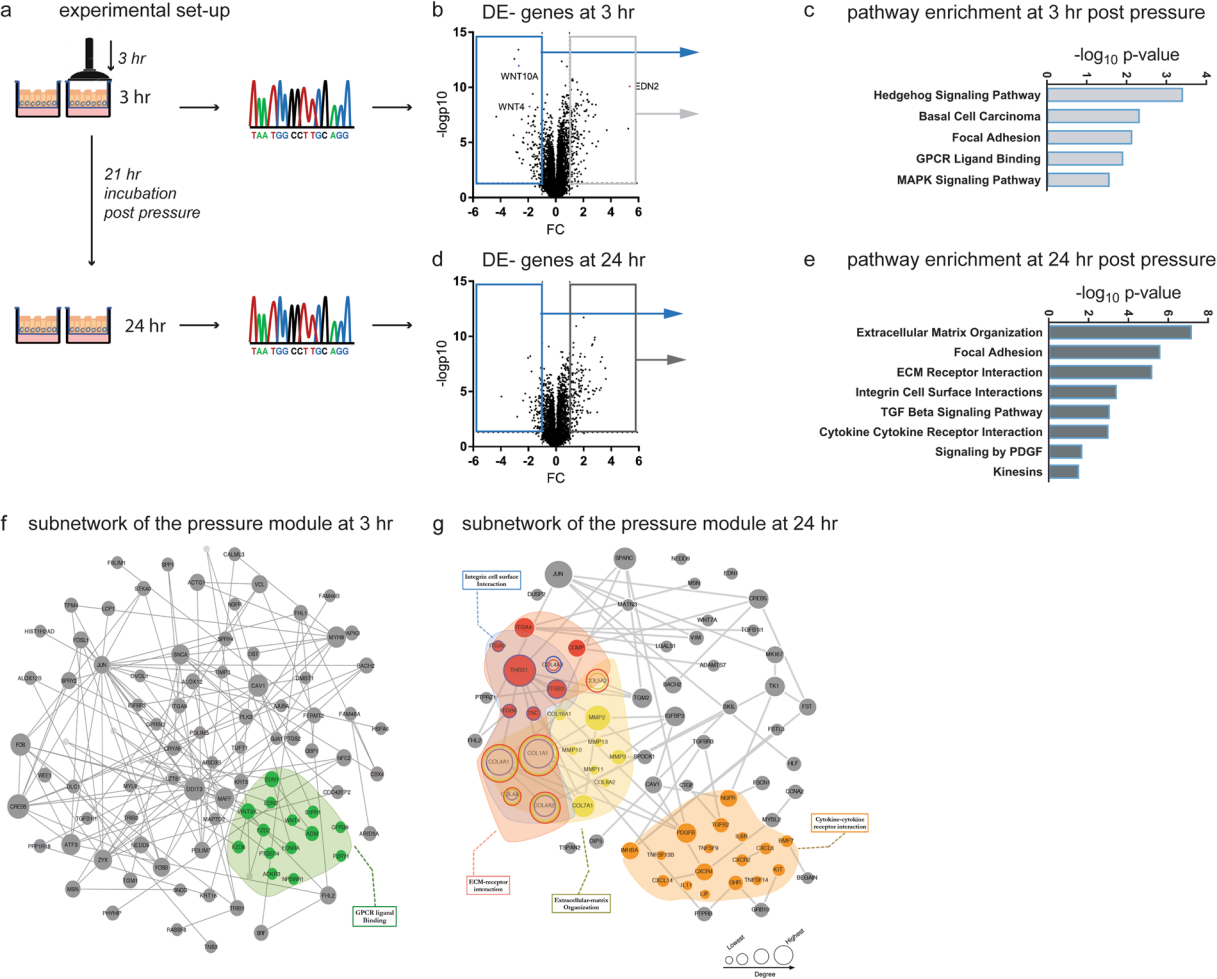
Compression induced molecular changes in HBECs. (**a**) Workflow of the methodology used to describe the overlapping and aligning expression signature between healthy HBECs exposed to compression and asthmatic HBECs at baseline. Expression data are collected and then, differentially expressed genes and miRs are mapped to the human interactome, resulting in an early and a late compression disease module. These were compared with the asthma disease module generated in Fig. 1. **(b)** Volcano plot visualizing DE genes at 3 hr post pressure application and pathways enriched in the early compression module. **(c)** Volcano plot visualizing DE genes at 24 hr post pressure application **(d)** and pathways enriched in the late compression module. **(e)** Visualization of the early **(f)** and late **(g)** pressure subnetworks. Activated pathways, overlapping with the asthma subnetwork are highlighted in colour.

The asthma module described above was determined in the absence of compression and then compared to gene expression profiles occurring in non-asthmatic cells exposed to compression. Specifically, we analysed gene expression induced soon after compression (the 3 hr time point) and later (the 24 hr time point). Control (non-asthmatic) treated cells for each donor and time point were used to normalize gene expression (Fig. 3a). Differential gene expression for each time point was assessed and the respective “compression modules” were defined. The same fold change (FC) cut-offs used for compression module description (FC 3 hr: 1.87 and FC 24 hr: 1.67) were applied to characterize alterations in gene expression. Within 3 hours of compressive stress, expression of 343 genes was altered (upregulated: 200; downregulated: 143; Fig. 3b). Pathway analysis performed by mapping all DE genes revealed alteration of the developmental Hedgehog pathway (Hedgehog signalling, adjusted p-value = 3.55e-07), the focal adhesion pathway (p-value = 4.32e-06), the G-protein coupled receptor signalling pathway (GPCR ligand binding, p-value = 1.12e-05), and the MAPK signalling pathway (p = 2.46e-05) (Fig. 3c–f and Table 2). The group of down regulated genes is associated with growth and development, including wingless proteins (*WNT*)^28^ and bone morphogenic protein (*BMP*) 7^29,30^ (Supplementary Fig. S3a–d). HBECs responded with the strong induction of genes associated with wound healing, matrix remodeling and epithelial repair, including adrenomedullin (*ADM*)^31^, osteopontin (*SPP1*)^32^, zyxin (*ZYX*)^33^ and tenascin (*TNC*)^34^. Subjecting the upregulated genes to gene ontology analyses for cellular compartments revealed an enrichment in focal adhesions (GO:0005925) and the cytoskeleton (GO:0005856) as summarized in Table 3.

**Table 2:** Pathways enriched immediately after compressive stress. (3 hr).

	Pathway	adj. p-value^a^ (Bonferroni)	genes in pathway
1	Hedgehog signalling	3.55E-07	RAB23, GLI3, WNT3A, GAS1, BMP7, WNT4, BMP2, WNT10A
2	Focal adhesion	4.32E-06	CAV1, ACTG1, ITGB3, ITGA9, PDGFA, TNC, THBS4, MYL9, VCL, ZYX, JUN, SPP1
3	GPCR ligand binding	1.12E-05	P2RY1, GPR39, PTGER4, WNT3A, ACKR3, ADM2, ADM, FZD8, WNT4, EDNRA, EDN1, EDN2, WNT10A, FZD2, S1PR5, NPBWR1, S1PR1
4	MAPK signalling pathway	2.46E-05	DUSP6, DUSP8, DDIT3, CACNA2D2, DUSP5, PDGFA, FOS, SRF, RASA2, JUN, HSPA6, FGF1, DUSP9

^a^Adjusted p-value: p-value was calculated using the Fisher exact test and adjustment was done using Bonferroni correction.

**Table 3:** Gene ontology analysis of genes immediately induced after compression.

Term	adj. p-value^a^	Genes
Focal Adhesion (GO:0005925)	9.15E-10	DST, TPM4, FBLIM1, ITGB3, TGFB1I1, FHL1, SPRY4, FHL2, PLAUR, TNC, MSN, RHOB, CSRP1, PALLD, DLC1, MYH9, LCP1, FERMT2, PDLIM7, VCL
Cytoskeleton (GO:0005856)	2.23E-04	DST, TPM4, MSN, NUAK1, KRT17, PALLD, CDC42EP2, MYH9, SPRY2, STK38L, LCP1, PDLIM5, NES, ULBP1, PDLIM7, VCL
Actin Cytoskeleton (GO:0015629)	4.40 E-3	TPM4, PALLD, DLC1, MYADM, MYH9, STK38L, LCP1, PDLIM5, ULBP1, PDLIM7
Contractile Actin Filament bundle (GO:0097517)	6.15 E-03	TPM4, FBLIM1, MYH9, LCP1
Stress Fibre (GO:0001725)	6.15 E-03	TPM4, FBLIM1, MYH9, LCP1
Actomyosin (GO:0042641)	1.02 E-02	TPM4, FBLIM1, MYH9, LCP1

^a^Adjusted p-value: p-value was calculated using the Fisher exact test and adjustment was done using Bonferroni correction.

**Figure 4:**
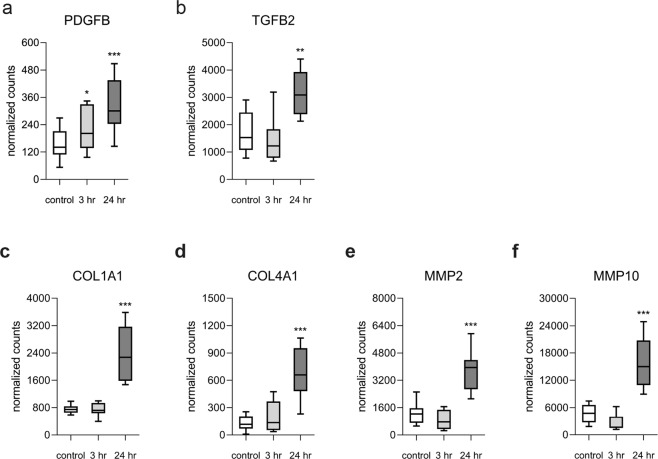
Compression induced remodeling associated genes. Genes, related to fibrotic responses, are elevated at 24 hr post compression. This list includes the soluble factors **(a)** platelet-derived growth factor β (PDGFB) and **(b)** transforming growth factor**-**β2 (TGFB2). As downstream targets of the fibrotic response we highlight **(c)** Col1a1, **(d)** Col4a1, **(e)** matrix metalloproteinases MMP-2 and **(f)** MMP-10. Values summarize the expression levels for n = 8 independent samples per group. The box and whisker plots represent the minimum, 25th percentile, median, 75th percentiles and the maximum. *p < 0.05 compared to control was considered significant.

This prompt reaction of the epithelium subsequently transitioned into a strong repair/fibrotic response at 24 hr post-compression. Among the 512 DE genes (upregulated: 294; downregulated: 220) (Fig. 3d,e), were those coding for the pro-fibrotic factors *TGF-β* and *PDGF-β* (Fig. 4a,b), various collagen chains and matrix metalloproteinases (MMPs) (Fig. 4c–f). We validated single molecules in independent samples using real-time RT-PCR (Supplementary Fig. S4). These molecules and pathways have been described in chronic disease conditions and had previously been thought to emerge as a result of a chronic inflammatory response (Fig. 3g and Table 4).

**Table 4:** Pathways enriched after compressive stress. (24 h).

	Pathway	adj. p-value^a^	genes in pathway
1	Extracellular matrix organization	6.03E-11	COL4A3, COL4A2, COL16A1, MMP3, COL4A1, MMP11, MMP13, COL4A4, COL1A1, MMP2, MMP10, COL5A2, COL8A2, COL7A1
2	Focal adhesion	2.30E-09	COL4A2, CAV1, COMP, COL4A1, ITGB3, ITGB6, ITGA9, PDGFB, THBS1, TNC, COL1A1, COL4A4, PDGFD, MYL9, JUN, COL5A2, ITGA4, ACTN1
3	ECM receptor interaction	5.84E-09	COL4A2, COL4A4, COMP, COL4A1, ITGB3, ITGA9, ITGB6, THBS1, TNC, COL1A1, COL5A2, ITGA4,
4	Integrin cell surface interaction	3.53E-07	COL4A3, COL4A2, COL4A4, COL4A1, ITGB3, ITGA9, ITGB6, THBS1, TNC, COL1A1
5	TGFbeta pathway	7.88E-07	SMAD7, LTBP1, COMP, THBS1, INHBA, BMP6, BMP7, TGFB2, CDKN2B, FST
6	Cytokine- cytokine- receptor interaction	9.01E-07	CXCL8, CXCR2, KIT, TNFSF13B, IL11, INHBA, TNFSF9, PDGFB, IL6R, TNFSF14, CXCR4, CXCL14, BMP7, LIF, TGFB2, NGFR, GHR
7	PDGF pathway	1.88E-05	COL4A3, COL4A2, COL4A4, COL4A1, PDGFB, THBS1, COL1A1, PDGFD, COL5A2, CAMK4,
8	Kinesins	2.72E-05	KIF4A, KIF15, KIF2C, KIF11, KIFC1

^a^Adjusted p-value: p-value was calculated using the Fisher exact test and adjustment was done using Bonferroni correction.

In addition to extracellular matrix proteins, compressive stress induced the expression of soluble immune modulatory factors, including 4-1BB (*TNFSF9*; FC: 1.75) and LIGHT (*TNFSF14*; FC: 2.12) in the later phase (Supplementary Fig. S5a,b). These data provide evidence for the ability of compressive stress in the absence of inflammation to enforce pronounced gene alterations in non-asthmatic HBECs.

### Compressive stress induces a disease signature resembling asthma

**Figure 5:**
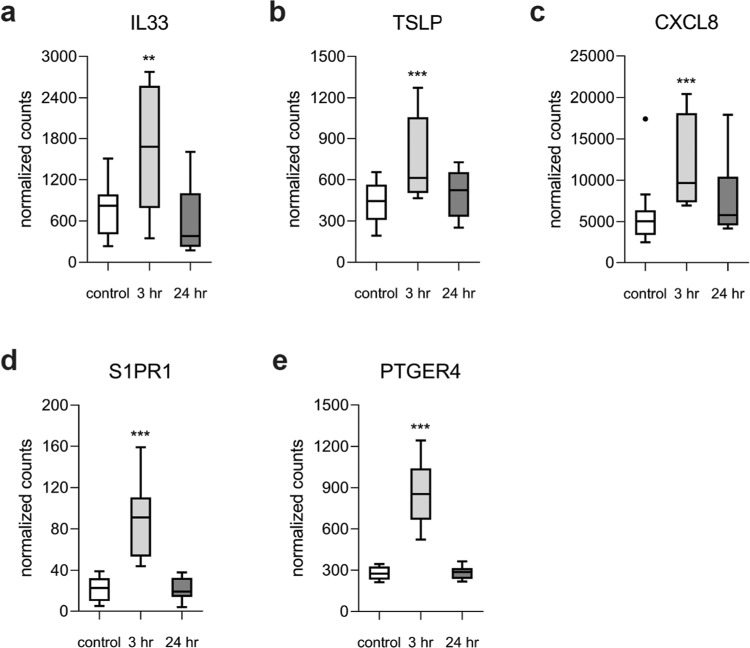
Compression induced alarmins and Th2-promoting mediators in healthy non-asthmatic HBECs. Compression on HBECs immediately (3 hr) induced the expression of **(a)** IL33, **(b)** Th2-promoting thymic stromal lymphopoietic protein (TSLP) as well as Cxcl8 **(c)**. This response was accompanied by immediate increase in lipid-mediator receptors **(d)** S1PR1 and **(e)** Prostaglandin E Receptor 4 (PTGER4). Values summarize the expression levels for n = 8 independent samples per group. The box and whisker plots represent the minimum, 25th percentile, median, 75th percentiles and the maximum. *p < 0.05 compared to control was considered significant.

To address the question of whether compressive stresses might initiate an immune response as observed in asthma, we assessed the expression level of epithelium derived Th2-promoting factors in non-asthmatic HBECs exposed to compression. Epithelial alarmins, including *IL-33* (Fig. 5a), thymic stromal lymphopoietin (*TSLP*; Fig. 5b) as well as *CXCL8* (Fig. 5c) were strongly induced in HBECs from non-asthmatic donors immediately after mechanical stimulus and receded at the 24 hr time point. Induction of *IL-33* and *IL-8* were validated in independent samples using real-time RT-PCR (Supplementary Fig. S6a,b).

In non-asthmatic HBECs, compressive stress promptly (3 hr time point) induced a set of genes mapping to ligands and receptors coupled to G-proteins in the asthma module. These included *S1PR1* and prostaglandin E receptor (*PTGER*) 4 (Fig. 5d,e). Real-time RT-PCR confirmed the induction of *PTGER4* expression in compressed epithelial cells (Supplementary Fig. S6c). Genes induced at 24 hr post-compression cover the area of the asthma module associated with matrix remodeling and map to extracellular –matrix organization, ECM-receptor interaction and Integrin-cell surface interaction (yellow, blue and red highlighted pathways (Fig. 3g).

These findings support the hypothesis that mechanical stimuli can induce disease-relevant gene expression in non-asthmatic HBECs and do so in the absence of prior inflammatory stimuli.

## Discussion

We report here that application of mechanical compression —akin to that which occurs during bronchospasm—is sufficient to evoke far-reaching molecular changes in human bronchial epithelial cells. We show, further, that these changes merge into an asthma-like molecular phenotype. Over time, initial transcriptional differences between non-asthmatic and asthmatic HBECs gradually faded and aligned to a similar molecular signature, comprising the induction of remodeling associated genes. Compression induced a prompt expression of inflammatory mediators promoting a type 2 inflammatory response. In non-asthmatic HBECs, early alterations in the compression response translated into a long-term repair/fibrotic response.

The airway epithelial lining forms a continuous tight barrier to protect the host from inhaled irritants and invading pathogens. Upon insult, epithelial cells become activated, secrete alarmins and pro-inflammatory molecules to recruit accessory immune cells and to induce proliferation and polarization programs aiming to restore epithelial integrity^35^. The asthmatic HBECs used in this study display a stable activated gene expression profile at baseline, which suggests a stable transcriptional program in these cells. It is unclear if a genetic or epigenetic signature determines the activated profile of asthmatic cells. Future studies might be directed towards the identification of molecular mechanisms to determine inherent differences between non-asthmatic and asthmatic airway epithelial cells and whether mechanical stimuli regulate any of these events. However, this work represents only the compression and asthmatic response of well-differentiated HBECs *in vitro*, so we cannot rule out that additional factors come into play *in vivo*. Furthermore, extending these approaches by capturing the dynamics of the cellular responses initiated by compressive stress and how these are propagated into an asthmatic phenotype will shed light on mechanisms which remain hidden by analysing only static networks. Analysing further biological information from other omics such as miRNA, proteomics and metabolomics, and integrating these, with the existing gene expression data will likely enhance the knowledge of how asthma can manifest at the airway epithelium.

In previous studies analysing the compression mediated response in HBECs only single genes have been described, while there has not been a network-based approach integrating the early events occurring upon application of acute compression to an asthma phenotype. Here we used HBECs from non-asthmatic and asthmatic donors to assess the functional consequences of compression on normal airway epithelial cells. Despite the small sample size, with which marginal differences due to high variability in gene expression were expected, we were able to detect marked differences in gene signature between HBECs from non-asthmatic and asthmatic donors at baseline as depicted in the asthma module, that overlapped with previously reported asthma-related genes and pathways in both human and mouse^2,36–38^. To further address the issue of relatively few asthmatic and mostly female donors in our experiment, we compared our retrieved disease signature, DE gene expression between asthmatic and non-asthmatic HBECs at baseline, with published asthma GEO datasets and calculated the overlap in DE-gene signature. The datasets were chosen to reflect the experimental set up of ALI-cultures (GSE37693) as well as the *in vivo* condition by analysing bronchial brushings isolated from severe asthmatics (GSE63142). In latter data set, both male and female donors were included. In GSE37693, stimulation of non-asthmatic HBECs with IL-13, mimicking the impact of Th2-inflammation on HBECs, induced a gene signature, that significantly overlapped (60.1%) with our asthma module. Even in epithelial cells isolated from severe asthmatic patients, a significant overlap (37%) in gene expression was observed (Fig. 2m).

The goal of this network approach was to map out the connectivity structure between genes that are affected by disease or perturbation (compressive pressure), instead of aiming for the identification of single differential expressed genes. For this, DE- gene information was used to generate the disease and pressure modules. In order to not apriori exclude genes and thus allow a more holistic view, a minimum p-value of 0.05 was used as a cut-off^20^. We validate this approach by retrieving many known genes based on the literature and our GEO confirmation analysis. To capture the molecular response, two time points were chosen to reflect initial inflammation and later remodeling. Besides the previously described autocrine and paracrine acting growth factors, including TGF-β^39–41^ and HB-EGF^42^, we describe multiple pathways initially affected by mechanical stimuli (Tables 2 and 3). A strong suppression of developmental genes, including Wnt and BMP proteins (Supplementary Fig. S3), was detectable promptly after compression and was accompanied by a marked induction of epithelial alarmins, including *IL-33* and *TSLP*. While a supportive activity of these factors has been documented for a variety of accessory immune cells^43,44^, IL-33 and TSLP are central for example to the initiation and enhancement of type 2 response typically seen in asthmatic inflammation^45–48^. Following compression (3 hr), an increased release of these factors from airway epithelial cells could activate type 2 innate lymphoid cells (ILC2) and augment type 2 inflammation in the lung^43,48,49^.

Increased expression of *PTGER4* and *S1PR1* further support the pro-inflammatory and immune-modulatory outcome. Signalling via *PTGER4* stimulates the activation of PKA via cAMP and induces transcription of CREB-dependent genes^50^. As a downstream target, we could detect increased *IL-11* expression at 24 hrs post-compression (Supplementary Fig. S7)^51^. IL-11 is essential for allergic sensitization, inflammation and airway remodeling in mice^52^ and reported to drive fibrotic responses, including cardiac and renal fibroblasts in humans^53^. Sphingolipid levels are elevated in the lungs of patients with allergic asthma. Specific blockade of sphingosine kinase 1 attenuates airway inflammation and hyper reactivity in mice^54^ further supporting the pro-inflammatory role of S1P signalling in bronchial epithelial cells.

In this acute compressive stress response, an induced expression of *FOS* and *JUN*, which together form the AP-1 transcription factor implicate a longer lasting change in protein expression post the analysis time points^55^. The later response is marked by an increased expression of several collagen chains as well as matrix metalloproteinases, accompanied by elevated pro-fibrotic factors TGF-β and PDGF-β. Taken together we observed a gradual alignment of the compression response with the asthma module, linking both phenomena with each other.

This analysis represents the first attempt in any biological system to understand the impact of mechanical forces on disease pathways using a network approach. The distinct activated profile of asthmatic epithelial cells suggests an “imprinted” signature in the absence of inflammation. It remains unclear, however, if a single compression may suffice to prime HBE cells for future reactions or if a repetitive stimulus would be needed to imprint an inflammatory signature as observed in asthmatic HBECs at baseline. The secretory activity of asthmatic HBECs at baseline could provide survival signals for resident innate and adaptive immune cells in the lung post-inflammation. Thus far it is postulated that the presence of antigenic molecules at the site of inflammation could activate survival programs in tissue resident memory immune cells and retain these in the previously inflamed organ^56,57^. Data provided here support the notion of a fundamental role of the airway epithelium in the control of local immune responses (Supplementary Fig. S8).

In the previous studies we have shown that compressive stress applied to HBECs induces a transition of the epithelial layer from a solid-like, immobile, jammed phase to a fluid-like mobile, unjammed phase, which is termed an unjamming transition^5,58,59^. This unjamming transition is reflected in the shape and mobility of the cells^5,58,59^. While the molecular mechanisms underlying this unjamming transition are not understood, the gene ontology analysis performed in this study provides new insights into the potential molecular mechanisms. For example, when mechanical compression caused cell layer unjamming the genes that were upregulated included those associated with focal adhesions and cytoskeleton (Table 3), which are responsible for internal force generation and maintaining cell shape. It remains unclear, however, as to whether the identified genes are the cause of the unjamming transition or the effect.

## Material and Methods

### Primary human bronchial epithelial cells

Primary HBECs were cultured in air-liquid interface (ALI) conditions as previously described^5,7,60–62^. We used HBECs from 4 donors with no pre-existing chronic lung disease (from here on referred to as non-asthmatic) and 4 asthmatic donors (Supplementary Table 1). HBECs were obtained at passage 0 or passage 1 from the Marisco Lung Institute at the University of North Carolina, Chapel Hill. Passage 2 cells were plated on transwell inserts coated with type I collagen and grown under submerged conditions for 5-6 days until the cells reached confluence. For each donor, two samples were introduced into the polarization process. Since every polarization runs independently for each well, these samples were considered as independent samples. To initiate ALI culture, apical media were removed and only basal media were subsequently fed every 2 days for additional 15 days, where the cells were well-differentiated with the appearance of basal, goblet, and ciliated cells (Supplementary Fig. S1)^61^.

Human lungs unsuitable for transplantation, including two cases of fatal asthma and two with asthma in the medical social history, were obtained from either Carolina Donor Services (Durham, NC), the National Disease Research Interchange (Philadelphia, PA), or the International Institue for Advancement of Medicine (Edison, NJ) under protocol #03-1396 approved by the University of North Carolina at Chapel Hill Biomedical Institutional Review Board. Informed consent was obtained from authorized representatives of all organ donors.

### Mechanical compression of HBECs

During asthma exacerbations, the excessive smooth muscle contraction causes the airway wall to buckle^63^. In the original development of mechanical compression model, Drazen and colleagues first used finite element analysis to compute the degree of compressive stresses imposed on the buckled airway epithelium by bronchospasm^64^. The estimated mechanical forces during maximal bronchospasm, is approximately 30 cm H_2_O. Using those computations as a guide, this team then went on to test the effect of mechanical compression with 30 cm H_2_O empirically in ALI culture of bronchial epithelial cells so as to optimize epithelial mechano-transduction responses^9^. Subsequently, this same apico-to-basal mechanical compression, was found to induce events that occur in the remodeled asthmatic airway, including increased matrix deposition^62^, goblet cell hyperplasia, and airway smooth muscle hyperplasia and hypercontraction, as well as production of asthma-associated mediators, including maspin, YKL-40, and tissue-factor positive extracellular vesicles^7,9,13,61,65–67^.

Well-differentiated HBECs cultured from non-asthmatic or asthmatic donors were mechanically compressed on ALI day 14^5,7,61,62^. At 20 hours prior to initiation of compression, cells were starved of bovine pituitary extract and epidermal growth factor. Cells were then exposed to 0 or 30 cm H_2_O of apical-to-basal transcellular pressure for 3 hours, thus mimicking the mechanical compression that occurs during bronchospasm^3^. Both control and compressed HBECs were harvested either immediately at 3 hr post compression, immediately after releasing pressure or at 24hr-post compression initiation, including an incubation period of 21 hours post pressure release. Harvested cells were used for isolation of RNA.

### RNA-Isolation, Library preparation and RNA sequencing

Total RNA was isolated organically using QIAzol lysis reagent and the Qiagen miRNeasy Kit (Qiagen). Quality was assessed using the Nanodrop 8000 spectrophotometer. Sequencing libraries were constructed with the TruSeq® Stranded Total RNA Library Prep Globin Kit (Illumina). Sequencing was performed using a HiSeq. 2500 instrument (Illumina). Trimmed reads were mapped to The GRCh38 reference genome using STAR^68^. Read counts were computed with htseq.^69^. Data were normalized and analysed with DESeq. 2^70^ with an FDR < 0.05.

### Gene ontology analysis

To describe the immediate changes induced by compression in BECs, we performed functional annotation analysis for upregulated genes. We used the Enrich^71^ method to obtain enriched GOCC terms.

Gene expression data for ALI-grown bronchial epithelial cells stimulated with IL-13 (GSE37693; n = 6 for each condition)^72^ and bronchial brushings from severe asthmatics (n = 56) compared to healthy controls (n = 27) (GSE63142)^73^ were retrieved from the GSE database.

### Protein-protein interaction network

To build a comprehensive human protein-protein interactome (PPI), we combined 15 databases with various kinds of experimental evidence that are currently available. The current updated human interactome includes 246,995 interactions connecting 16,706 unique proteins, which is more than 40% larger in number in comparison with our previously used human interactome^74^. Specifically, we focused on the high-quality PPIs with four types of data:

1. binary PPIs tested by high-throughput yeast-two-hybrid (Y2H) systems: we combined binary PPIs tested from two public available high-quality Y2H datasets^75,76^ and one unpublished dataset. This resource is available online at (https://ccsb.dana-farber.org/interactome-data.html).
2. kinase-substrate interactions by literature-derived low-throughput and high-throughput experiments from KinomeNetworkX^77^, Human Protein Resource Database (HPRD)^78^, PhosphoNetworks^79,80^, PhosphositePlus^81^, DbPTM 3.0^82^, and Phospho. ELM^83^.
3. carefully literature-curated PPIs identified by affinity purification followed by mass spectrometry (AP-MS), Y2H and by literature-derived low-throughput experiments, and protein three-dimensional structures from BioGRID^84^, PINA^85^, Instruct^86^, HPRD^78^, MINT^87^, IntAct^19^, and InnateDB^88^.
4. signaling network by literature-derived low-throughput experiments as annotated in SignaLink2.0^89^.

All data were downloaded in December 2015. The genes were mapped to their Entrez ID based on the NCBI database^90^ as well as their official gene symbols based on GeneCards (http://www.genecards.org/). Duplicated pairs were removed. Data from inferred data, such as evolutionary analysis, gene expression data, and metabolic associations were excluded.

### Quantitative real-time RT-PCR

Two μg of total RNA was used to synthesize cDNA using MultiScribe reverse transcriptase (Thermo Fisher Scientific), as described previously^65^. Quantitative real-time PCR was performed using iTaq Universal SYBR Green Supermix (BioRad). Following an initial denaturation step at 95 °C for 10 minutes, 40 cycles of PCR at 95 °C for 15 seconds followed by at 60 °C for 60 seconds were performed in a Bio-rad CFX96 real-time PCR detection system. Primers specific for *COL1A1* (5′-CAC ACG TCT CGG TCA TGG TA -3′/5′-AAG AGG AAG GCC AAG TCG AG-3′); *FN1* (5′-CCC CAT TCC AGG ACA CTT CT-3′/5′-TGC CTC CAC TAT GAC GTT GT-3′); *IL-8* (5′-CAC CGG AAG GAA CCA TCT CA-3′/5′-AGA GCC ACG GCC AGC TT-3′); IL-33 (5′- TGC ATG CCA ACA ACA AGG AA-3′/5′-AAG GAC AAA GAA GGC CTG GT-3′); (5′- ACT CTT TTG ATG GCC CAG GA-3′/5′- GAG TGG CCA AGT TCA TGA GC-3′); *MMP-10* (5′- ACT CTT TTG ATG GCC CAG GA-3′/5′- GAG TGG CCA AGT TCA TGA GC-3′); *PTGER4* (5′-TAC TCA TTG CCA CCT CCC TG-3′/5′-ATT CGG ATG GCC TGC AAA TC-3′) and *GAPDH* (5′- TGG GCT ACA CTG AGC ACC AG -3′/5′- GGG TGT CGC TGT TGA AGT CA-3′) were used to test the mRNA expression. PCR quantification was done with the 2^−ΔΔCT^ method, with normalization to Gapdh as the housekeeping gene. All standard procedures were performed according to the manufacturer′s instructions.

### Statistical analysis

Results summarize the RNA sequencing results of four individuals per group. Each individual was assessed in replicate and the average values were used for further analysis. All computations were performed with Python 2.7. Log_2_ transformed values were used to compare experimental groups with Mann-Whitney *U* test. Where appropriate, a Wilcoxon matched-pairs signed rank test was done. *p* < 0.05 was considered significant. We calculated the significance of the overlap of the here described “disease signature” and the DE-genes from publicly available data sets GSE37693 and GSE63142 using a gene set enrichment analysis.

## Supplementary information

Supplementary Information.

## Data Availability

The RNA-sequencing data that support the findings of this study are available upon request from the senior author of this article.
