# Supplementary material for: Mechanical forces induce an asthma gene signature in healthy airway epithelial cells
Source: Sci Rep. 2020 Jan 22;10:966. doi: 10.1038/s41598-020-57755-8 (PMC6976696; doi:10.1038/s41598-020-57755-8)
Supplement: Supplementary file 1 — Supplementary Information. [file 41598_2020_57755_MOESM1_ESM.pdf]

Mechanical forces induce an asthma gene signature in healthy airway epithelial cells.

Supplementary Information

Ayşe Kılıç, Asher Ameli, Jin-Ah Park, Alvin Kho, Kelan Tantisira, Marc Santolini, Feixiong Cheng, Jennifer Mitchel, Maureen McGill, Michael J. O'Sullivan, Margherita De Marzio, Amitabh Sharma, Scott H Randell, Jeffrey M Drazen, Jeffrey Fredberg, and Scott T. Weiss

Corresponding author:
Scott T. Weiss, MD, MS
Brigham and Women's Hospital
Channing Division of Network Medicine
181 Longwood Avenue
Boston, MA 02115
Email: scott.weiss@channing.harvard.edu

Supplementary Table 1. Donor characteristics

**non- asthmatic donors (ID)**	**Age**	**Sex**	**asthmatic donors (ID)**	**Age**	**Sex**
U7	16	F	UD2 (fatal)	28	F
U8	23	F	UD3 (fatal)	20	M
U9	59	F	UD4	59	F
U16	47	F	UD5	50	F

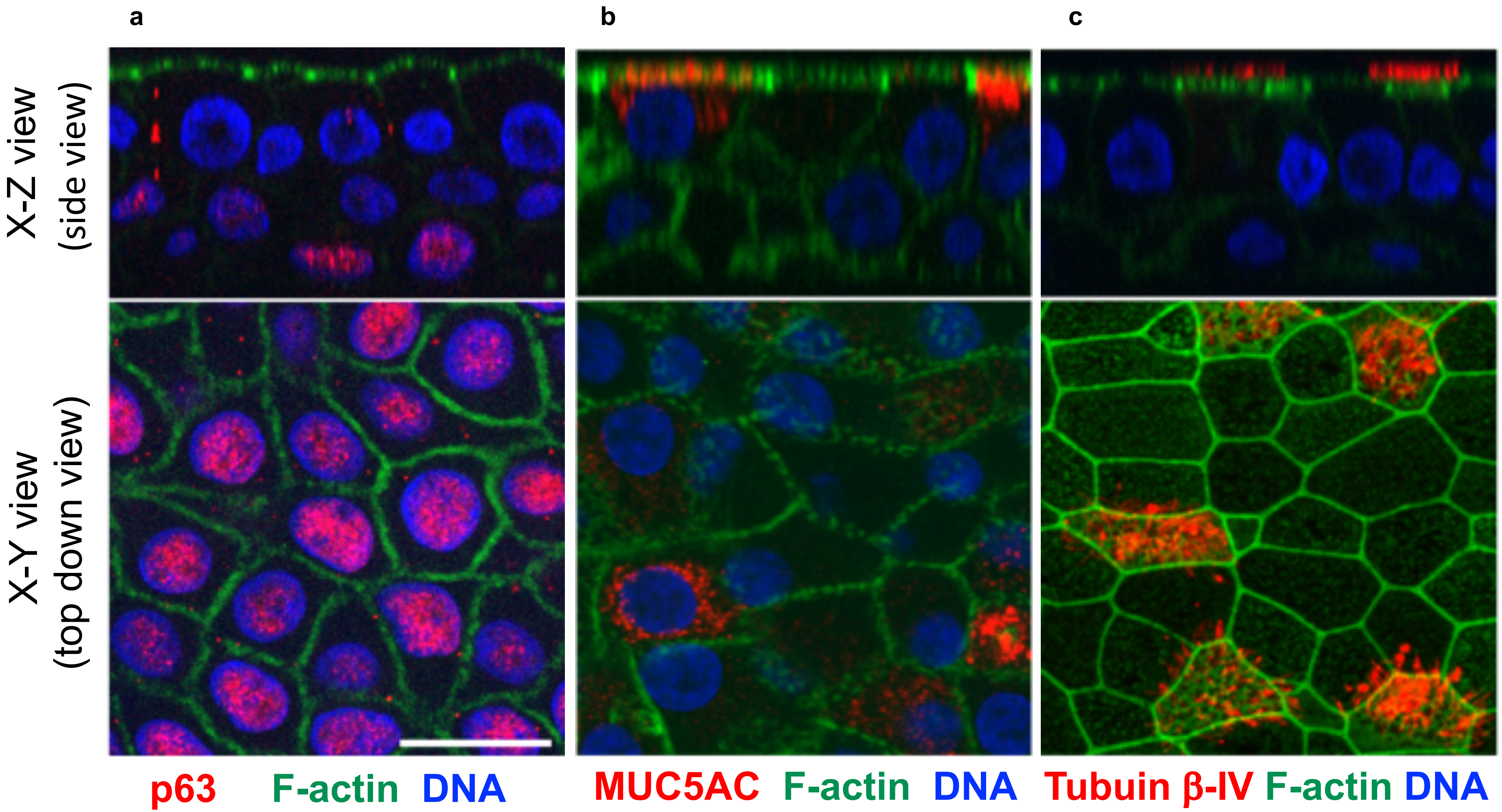

Supplementary Fig. S1. Air-Liquid Interface grown human bronchial epithelial cells. Well-differentiated HBE cells were stained for a basal cell marker, p63 (a), for a goblet cell marker, MUC5AC (b), and for a ciliated cell marker, Tubulin beta-IV (c). In all figures, red stains for epithelial cell specific markers, green stains for F-actin, and blue stains for nuclei. The scale bar is 20 μm.

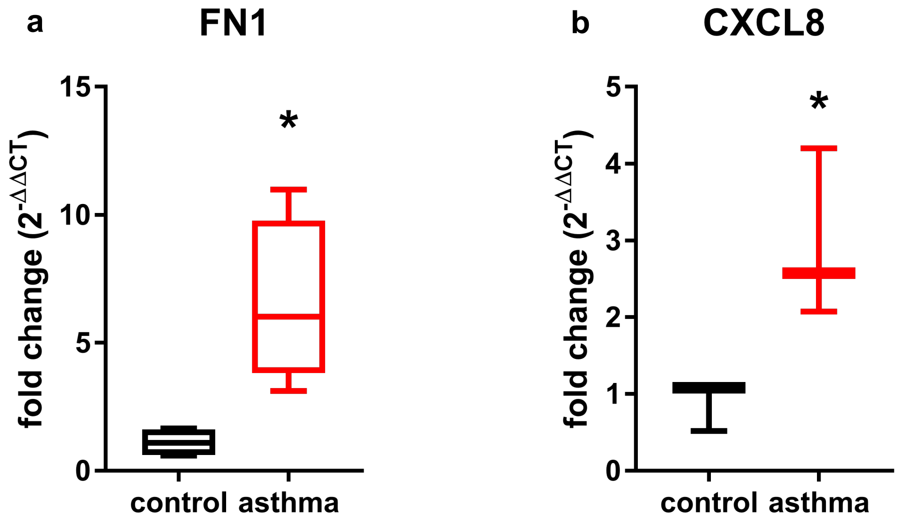

Supplementary Fig. S2. Baseline gene expression differences in asthmatic HBECs. RNA-expression for (a) FN1, (b) CXCL8 are highly expressed in asthmatic HBECs at baseline. Values summarize the expression levels measured by rela-time RT PCR and normalized to Gapdh expression for n=4 HBECS per group and were analyzed by Mann-Whitney *U*-test. *$p < 0.05$ was considered significant.

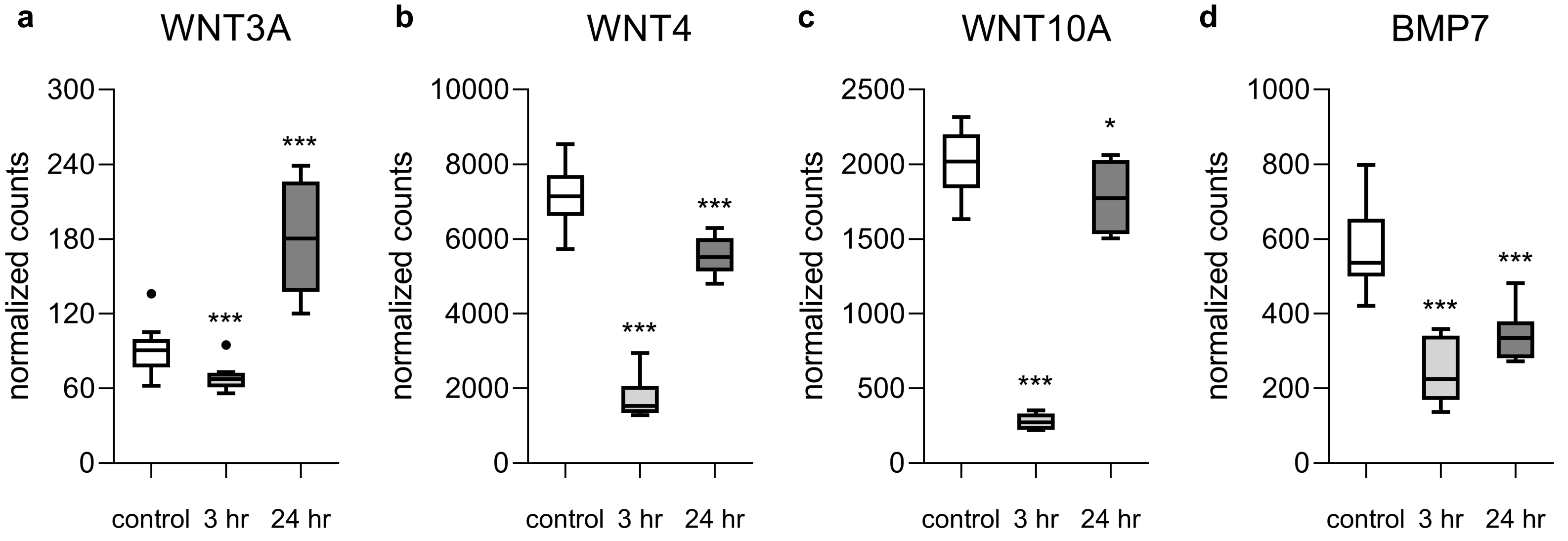

Supplementary Fig. S3. Increased airway pressure immediately suppresses the expression of developmental genes in healthy HBECs. RNA-expression for (a) WNT3a, (b) WNT4 and (c) WNT10a as well as (d) BMP7 at baseline and 3 hr and 24 hr after pressure application in healthy HBECs.Values summarize the expression levels for n=4 HBECS per group. The box and whisker plots represent the minimum, 25th percentile, median, 75th percentiles and the maximum. *padjust < 0.05 compared to control was considered significant.

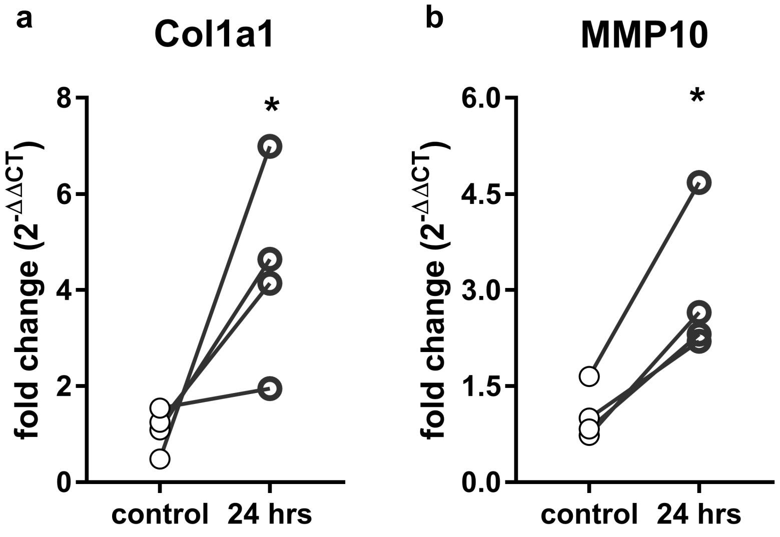

Supplementary Fig. S4. Increased airway pressure induces the extracellular matrix associated genes. RNA-expression for (a) Col1a1 and (b) MMP10 at 24 hr after pressure application in healthy HBECs. Values summarize the expression levels measured by rela-time RT PCR and normalized to Gapdh expression for n=4 HBECS per group and were analyzed by Wilcoxon matched-pairs signed rank test. *$p < 0.05$ was considered significant.

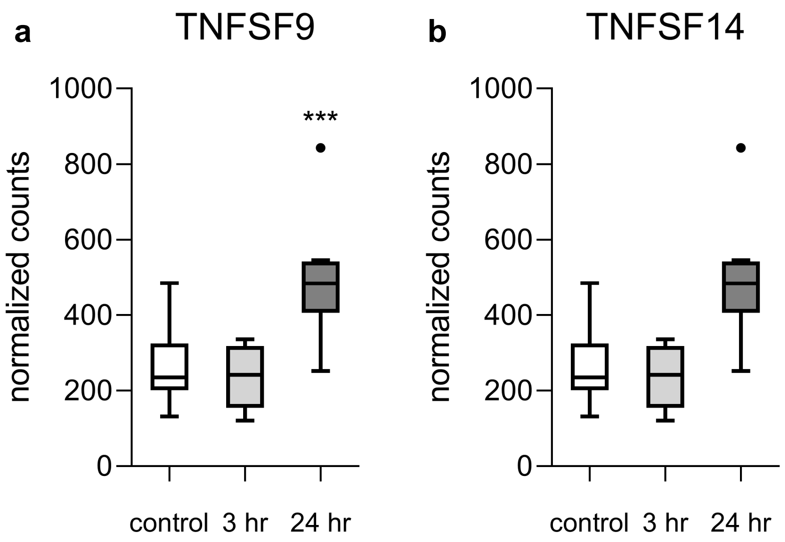

Supplementary Fig. S5. **Pressure induced expression of immunmodulatory factors.** In healthy HBECs exposed to increased pressure for 3 hr TNFSF9 (4-1BB-L) and TNFSF9 (LIGHT) IL-11 was detected at 24 hr. Values summarize the expression levels for n=8 HBECS per group. The box and whisker plots represent the minimum, 25th percentile, median, 75th percentiles and the maximum. *padjust < 0.05 compared to control was considered significant.

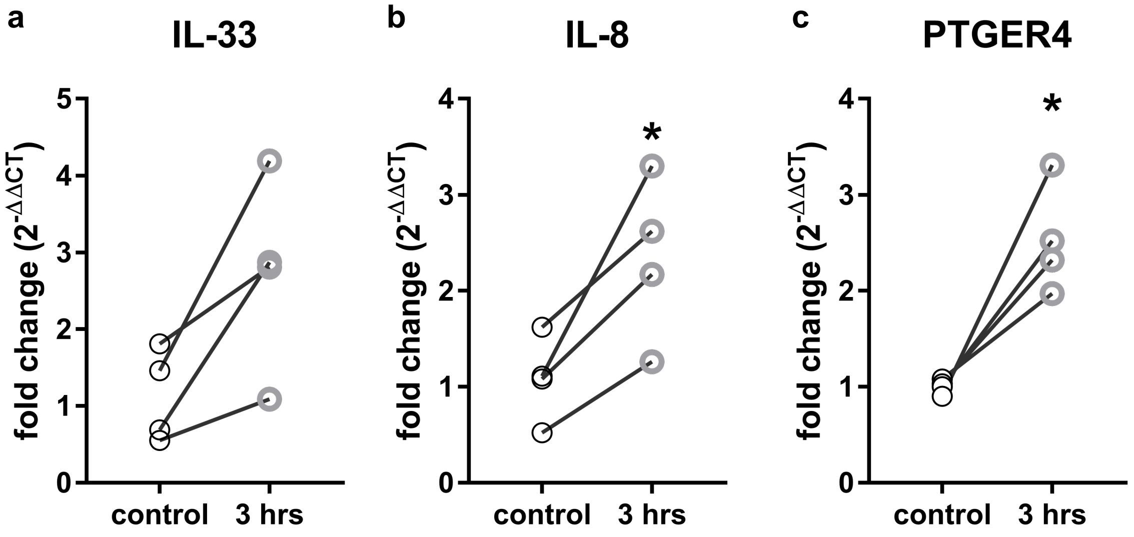

Supplementary Fig. S6. Increased airway pressure immediately induces the expression of immediate early genes. RNA-expression for (a) IL-33, (b) CXCL8 and (c) PTGER4 at 3 hr after pressure application in healthy HBECs. Values summarize the expression levels measured by rela-time RT PCR and normalized to Gapdh expression for n=4 HBECS per group and were analyzed by Wilcoxon matched-pairs signed rank test. *$p < 0.05$ was considered significant.

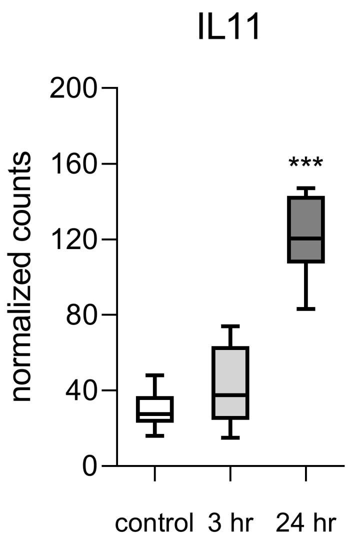

Supplementary Fig. S7. Pressure induced expression of pro-fibrotic IL-11. In healthy HBECs exposed to increased pressure for 3 hr IL-11 was detected at 24 hr. Values summarize the expression levels for n= 8 HBECS per group. The box and whisker plots represent the minimum, 25th percentile, median, 75th percentiles and the maximum. *padjust < 0.05 compared to control was considered significant.

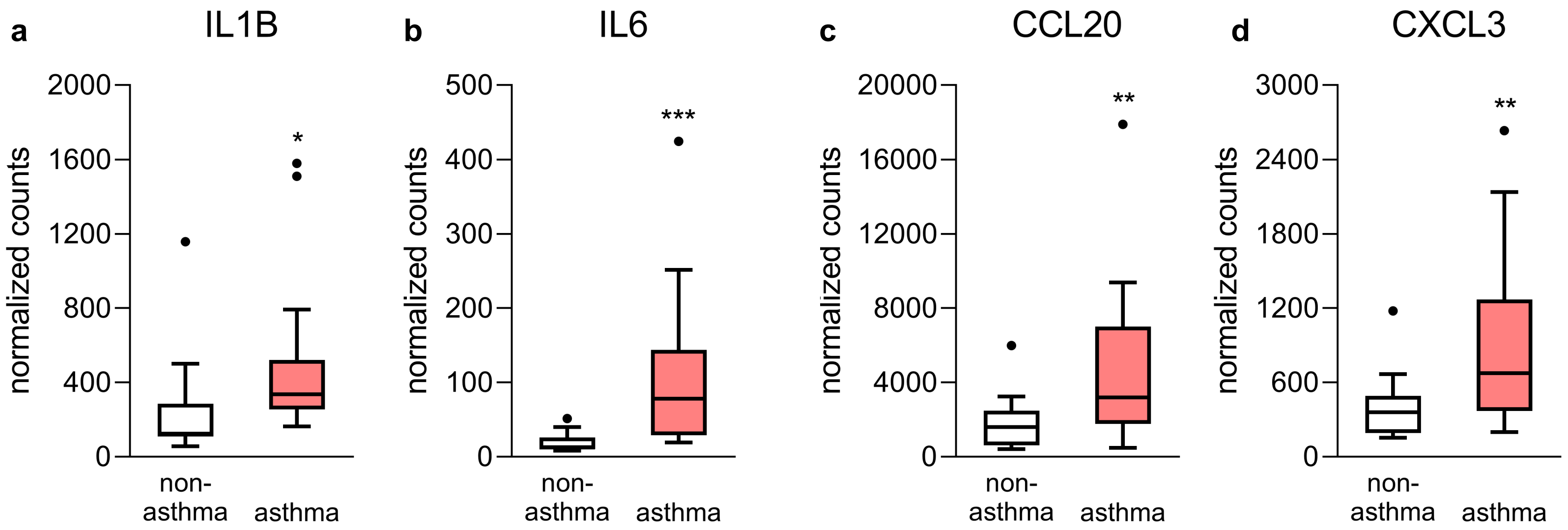

Supplementary Fig. S8. Bronchial epithelial cells from asthmatics express pro- inflammatory cytokines and chemokines at baseline. RNA-expression for (a) IL1β, (b) IL-6 and the chemokines (c) Ccl20 as well as (d) Cxcl3 at baseline in healthy and asthmatic HBECs. Values summarize the expression levels for n= 8 HBECs per group. The box and whisker plots represent the minimum, 25th percentile, median, 75th percentiles and the maximum. *padjust < 0.05 compared to non-asthma was considered significant.
